# Plastics in sea surface waters around the Antarctic Peninsula

**DOI:** 10.1038/s41598-019-40311-4

**Published:** 2019-03-08

**Authors:** Ana L. d. F. Lacerda, Lucas dos S. Rodrigues, Erik van Sebille, Fábio L. Rodrigues, Lourenço Ribeiro, Eduardo R. Secchi, Felipe Kessler, Maíra C. Proietti

**Affiliations:** 1 Instituto de Oceanografia, Universidade Federal do Rio Grande, Rio Grande, Brazil; 2 School of Environment and Life Sciences, University of Salford, Manchester, United Kingdom; 3 Institute for Marine and Atmospheric Research Utrecht, Utrecht University, Utrecht, Netherlands; 4 Centro de Estudos Costeiros, Limnológicos e Marinhos, Universidade Federal do Rio Grande do Sul, Imbé, Brazil; 5 Laboratoire Mer Molécules Santé, Institut Universitaire Mer et Littoral, Université de Nantes, Nantes, France; 6 Marine and Environmental Sciences Centre, Faculdade de Ciências da Universidade de Lisboa, Lisbon, Portugal; 7 Escola de Química e Alimentos, Universidade Federal do Rio Grande, Rio Grande, Brazil

## Abstract

Although marine plastic pollution has been the focus of several studies, there are still many gaps in our understanding of the concentrations, characteristics and impacts of plastics in the oceans. This study aimed to quantify and characterize plastic debris in oceanic surface waters of the Antarctic Peninsula. Sampling was done through surface trawls, and mean debris concentration was estimated at 1,794 items.km^−2^ with an average weight of 27.8 g.km^−2^. No statistical difference was found between the amount of mesoplastics (46%) and microplastics (54%). We found hard and flexible fragments, spheres and lines, in nine colors, composed mostly of polyurethane, polyamide, and polyethylene. An oceanographic dispersal model showed that, for at least seven years, sampled plastics likely did not originate from latitudes lower than 58°S. Analysis of epiplastic community diversity revealed bacteria, microalgae, and invertebrate groups adhered to debris. Paint fragments were present at all sampling stations and were approximately 30 times more abundant than plastics. Although paint particles were not included in plastic concentration estimates, we highlight that they could have similar impacts as marine plastics. We call for urgent action to avoid and mitigate plastic and paint fragment inputs to the Southern Ocean.

## Introduction

Plastics make up about 90% of marine litter^1,2^, and it is estimated that there are between 15 and 51 trillion plastic particles floating on the surface of the oceans^3^. Currently, plastics are widely distributed in the marine environment, in both hemispheres from the tropics to the poles, with accumulation zones along coastlines, the seafloor, and surface waters, especially in convergence zones such as vortexes and the center of subtropical gyres^2–6^. Marine plastics can have several impacts, such as degradation of habitats, impairment to navigation, contamination of environments, and direct effects on biota through ingestion, asphyxiation and entanglement; these direct effects have already been reported for at least 700 marine species^7–10^. Another worrying but still poorly understood effect of plastics in the marine environment is their role as an artificial substrate for the fixation of organisms^7^. The hydrophobic nature of plastics stimulates biofilm formation and allows the establishment of numerous organisms (“epiplastic” organisms)^11–13^ that constitute a new marine ecosystem called the “Plastisphere”^14^ that can harbor different groups including bacteria, viruses, fungi, micro and macroalgae, mollusks, cnidarians, crustaceans and fish^7,12,15,16^.

The impacts of epiplastic organisms on the marine environment can be diverse. For instance, they may increase consumer attraction for plastics when they perceive this colonized material as a food item, since the biofilm on its surface may smell and look like food^17^. Once ingested, plastics can obstruct or injure the gastrointestinal tract of animals, and possibly lead to death^10,18^. Exposure to plastics can also lead to a reduction (up to 45%) in the growth of microalgae^19^. In addition, the Plastisphere has already been shown to contain pathogenic organisms, such as *Vibrio* bacteria, which can cause diseases to both marine animals and humans^14^. Recently, Arias-Andres *et al*.^20^ analyzed aquatic microbial communities living adhered to plastics, and observed a greater transferal of plasmids carrying antibiotic resistance genes (via horizontal transfer) than in free-living communities. This may eventually transfer other genes that favor the establishment of new traits in bacterial communities by evolutionary changes.

The Plastisphere can be especially impacting in terms of global biological invasions, through the dispersal of species between environments and regions via surface water transport^7,12,14^. Many plastics that enter the oceans are less dense than seawater, and float^21^. Additionally, factors such as ultraviolet (UV) radiation and interaction with atmospheric O_2_ alter the physical and chemical properties of these materials, causing some plastics, originally denser than seawater, to decrease their density and float at the ocean surface^21,22^. By remaining at the surface, plastics can be transported via winds and currents over long distances and across ocean basins; the transoceanic dispersion of debris containing epiplastic organisms may cause changes in marine biogeographical patterns^16^. This has already been shown by Carlton *et al*.^16^, who report a transoceanic biological rafting event after the 2011 tsunami in Japan, with 289 species from 16 phyla crossing the Pacific Ocean from the Japanese coast to Hawaii and North America. The dispersal of plastics and their inhabitants to remote areas of the planet, such as polar regions, has also been reported^23–25^.

Antarctica, established as a World Heritage Site until 2041 and an international territory devoted to peaceful and scientific purposes^26^, has suffered strong environmental impacts in recent years. Such impacts include the acidification of the surrounding ocean due to increase in atmospheric CO_2_^27^ and the growth of marine plastic pollution^24,28,29^. All studies on marine debris in surface waters, beaches and the seafloor in Antarctica highlight that this problem is still poorly understood and requires further evaluation in order to develop tangible and efficient strategies to prevent and mitigate marine plastic pollution in this remote and sensitive environment^23,24,28,29^. Sources of plastics in Antarctica can be diverse, including direct sources via disposal or inadequate management of waste produced by ships and research stations^29^, and indirect sources such as transport by marine currents, which can carry plastics from distant areas located at lower latitudes^24,29,30^. Such varied sources can lead to a diversity in the types of plastics found in Antarctica; for example, plastic fragments, fishing lines and different plastic packages have been reported in this region^24^. An example of the impact of plastic debris to marine animals in Antarctica is entanglement, reported mainly for mammals and birds and having affected over a thousand fur seals from 1989 to 2008^24^. Ingestion of plastics, as well as presence of this material in nesting areas, is also a common problem^24^. In this manner, our study aims to determine the concentrations, characteristics, and origins of plastic debris in oceanic surface waters around the Antarctic Peninsula.

## Results

### Abundance and characteristics of plastics

**Figure 1:**
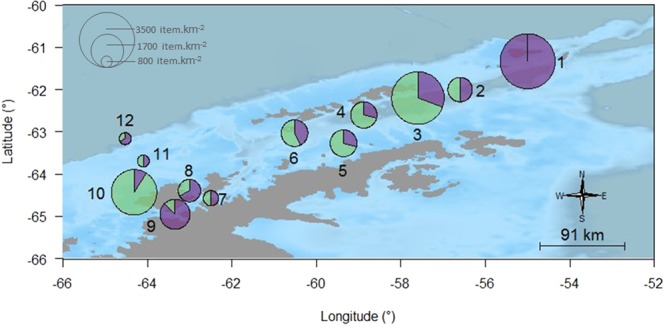
Abundance of micro (purple) and mesoplastics (green) per sampling point in Antarctic waters. The map was created using the *marmap* package and graphics were inserted using the *mapplots* package and *add*.*pie* function on *R* 3.5.0 (https://www.r-project.org).

We found 78 plastic items with different abundances and characteristics at the sampling stations around the Antarctic Peninsula (Table 1). Plastic debris were found in all surface trawls. The total average concentration of plastics for the area was estimated at 1,794 items.km^−2^, with maximum densities of respectively 3,524 items.km^−2^ and 3,474 items.km^−2^ at stations 3 and 1, and a minimum of 755 items.km^−2^ at station 12. The total weight of the 78 plastic pieces was 1.21 g and the average weight was estimated at 27.8 g.km^−2^, ranging from 0.21 g.km^−2^ at station 2 (weight of four plastic pieces) to 146 g.km^−2^ at station 8 (eight plastic pieces). Station 3 had the highest number of plastics (n = 13), which had an extrapolated weight of 50 g.km^−2^. When expressed in terms of volume, plastic concentration was 0.000132 g.m^−3^ and 0.008 items.m^−3^. We found no correlation between plastic abundance and environmental parameters (sea state, wind speed and local depth) at the sampling points (p > 0.05) (see Supplementary Fig. S1). The most common plastic format was ‘fragment’ (51.3%), with lower abundance of items in categories ‘line’ (42.3%), and ‘sphere’ (6,4%); in terms of flexibility, most items (83%) were categorized as flexible (Table 1). Fragments ranged from 1 mm to 67 mm; spheres ranged from 2 mm to 6 mm; and lines from 2 mm to 74 mm. There was an almost equal number of microplastics (54%) and mesoplastics (46%), and no macroplastics were found in this study. However, although there was no statistical difference between micro and mesoplastics (p = 0.69), we observed difference in the proportion of these two size categories between the sampling points, with microplastics predominating at sampling points 1 (more than 98% of plastics), 8, 9 and 12, and mesoplastics being more abundant at sampling points 3, 4, 5, 6 and 10. A more balanced proportion of the two size classes was found at stations 2, 7 and 11 (Fig. 1).

**Table 1:** Number and weight of plastics found in twelve sampling points at surface waters around the Antarctic Peninsula, according to shape and flexibility.

Station	Number of plastics	Weight (g)	Shape (n)			Flexibility (n)	
			Fragment	Line	Sphere	Rigid	Flexible
# 1	6	0.02743	3	3	—	—	6
# 2	2	0.00052	—	2	—	—	2
# 3	13	0.18478	8	4	1	2	11
# 4	7	0.03719	4	1	2	3	4
# 5	7	0.06932	6	1	—	—	7
# 6	7	0.06039	4	2	1	3	4
# 7	4	0.00089	1	3	—	—	4
# 8	6	0.19845	3	3	—	—	6
# 9	8	0.57977	3	5	—	—	8
# 10	11	0.03610	5	5	1	3	8
# 11	4	0.00801	2	2	—	1	3
# 12	3	0.00779	1	2	—	1	2

**Figure 2:**
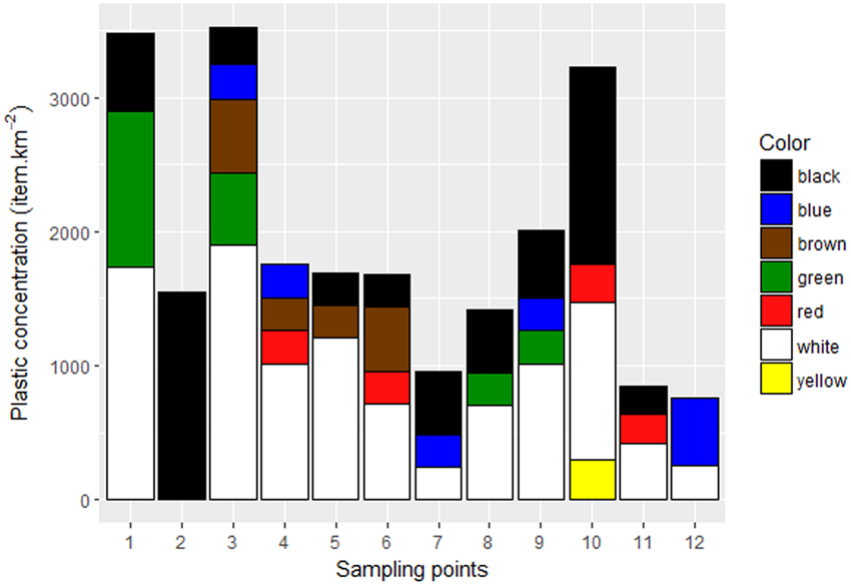
Concentrations and colors of plastics per sampling point off the Antarctic Peninsula.

In terms of color, the sampled plastics were white, yellow, blue, green, red, black and brown. The concentration of items, as well as the color of particles found at each sampling point, is shown in Fig. 2. The color white was the most abundant (47%), and white plastics were present in all sampling points except point 2. The second most common color was black (23%), which was also present in almost all points with the exception of 4 and 12. White and black composed more fragments than the other plastic shapes. The other colors represented lower proportions, with blue, brown and green representing the same percentage (7%), followed by red (5%) and yellow (1%). Point 3 had the largest diversity of colors (Shannon’s index = 1.304), while the lowest was observed at point 2 (Simpson’s index = 0.000), where only black plastics were found. Except for point 2, in general there was no dominance of a specific color in plastic items at sampling points, with point 6 presenting the highest equitability (that is, lower dominance) of different colors (Simpson’s index = 0.6939), while points 5 (Simpson’s index = 0.4489) and 12 (Simpson’s index = 0.4444) presented lower equitability between the colors. Polymer analysis of 28 items showed that most plastics were composed of polyurethane (35%), followed by polyamide (25%), polyethylene (21%), polystyrene (11%) and polypropylene (8%). The majority of fragments were composed of polyurethane, and most lines were composed of polyamide.

### Dispersal of plastics around the Antarctic Peninsula

**Figure 3:**
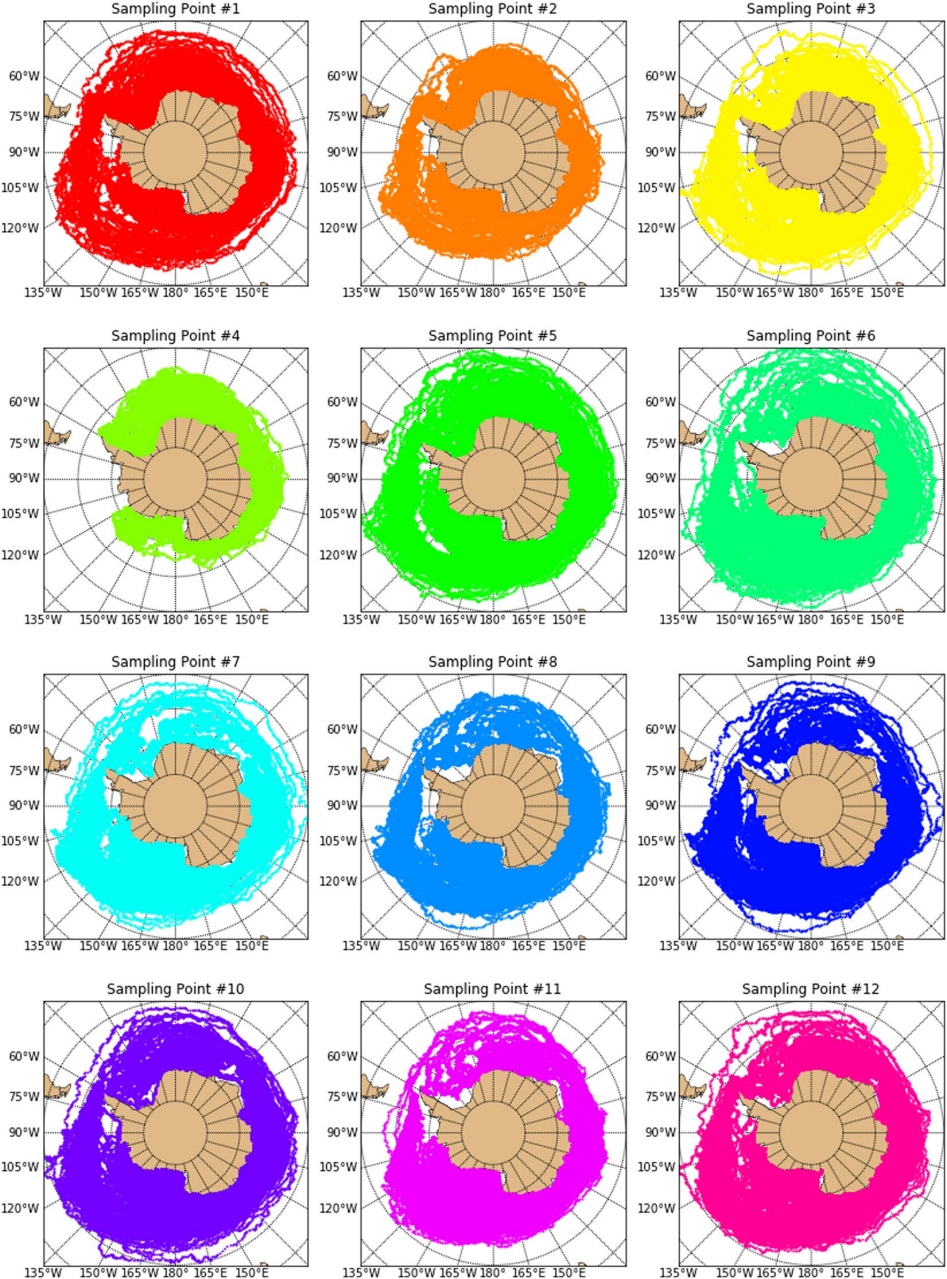
Dispersal model of plastic particles sampled with a manta net in surface waters of twelve points off the Antarctic Peninsula. The model backtracked seven years of dispersal, using ocean surface current data from HYCOM and Stokes drift data from WaveWatchIII.

Our backtracking dispersal model showed that the sampled plastics did not originate from latitudes lower than 58°S for at least the past seven years, indicating that they have likely been around the Antarctic continent during this time or entered the Southern Ocean more recently through local sources (Fig. 3, animation available in Figshare link: https://figshare.com/s/610834334d02b705abaf). During the modeling period (seven years before sampling in Feb 2017), it was observed that plastics sampled at most stations could have originated from anywhere poleward of 58°S off Antarctica; meanwhile, plastics sampled at station 4 presented more restricted sources closer to the continent (Fig. 3). The plastics sampled at the other localities presented more diffused origins throughout the Southern Ocean, and could have originated from any point around the Antarctic continent. We did not observe any major difference in terms of origins of plastics collected at the different points in the Southern Ocean.

### Paint particles

**Figure 4:**
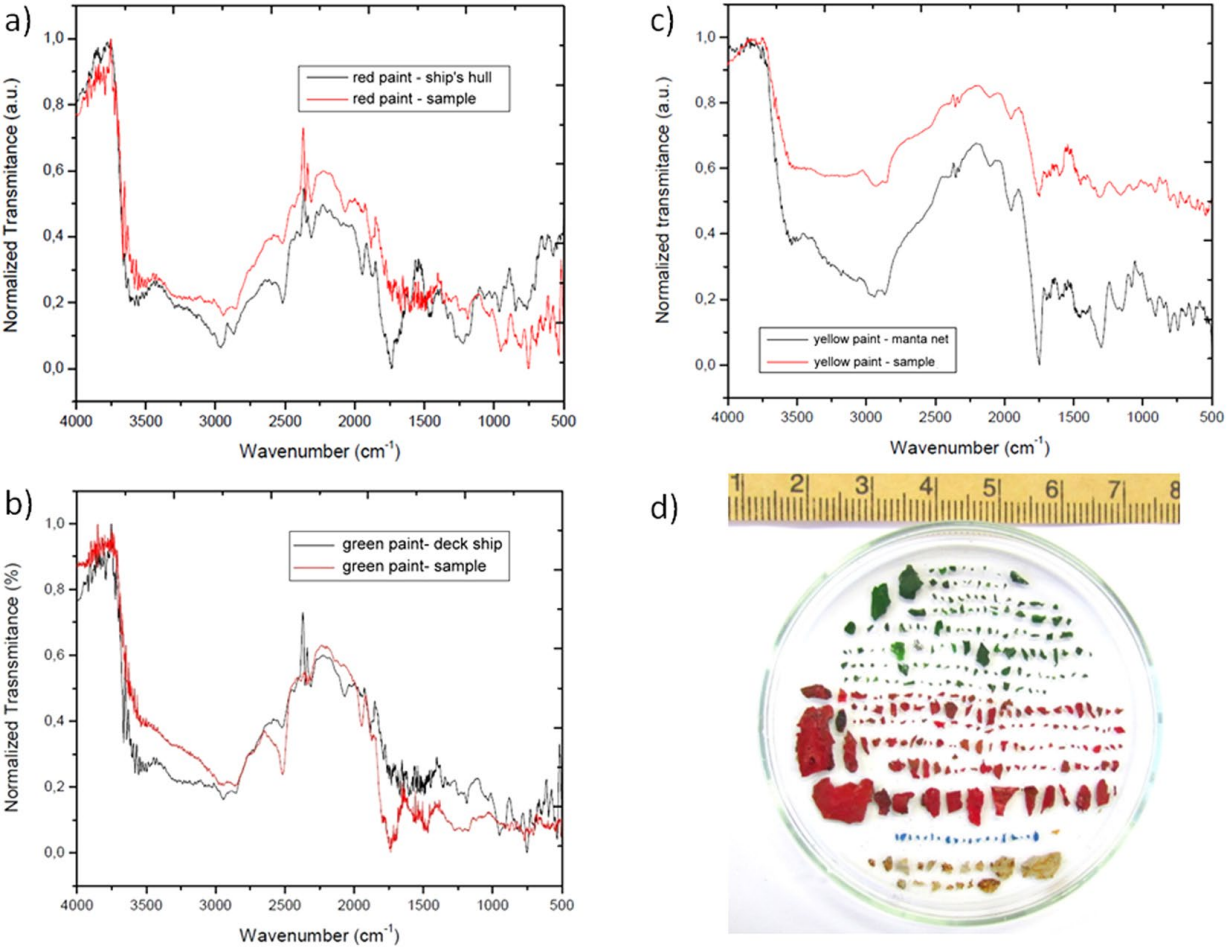
Fourier Transform Infrared (FTIR) spectra from (**a**) red, (**b**), green, and (**c**) yellow ocean paint fragments compared with those from the hull of the ship (red and green) and the manta net frame (yellow); (**d**) general appearance of paint pieces sampled around the Antarctic Peninsula.

We found a very high number of paint particles in the samples (n = 2805), ranging from 0.3 mm to 23 mm and in seven colors: red, green, yellow, blue, orange, gray and white (Fig. 4). Polymer composition analysis of 21 samples revealed that paint fragments were made of polyurethane, with varying degrees of degradation. To determine if part of the fragments were being released during sampling, we compared the spectra of red, green, and yellow paint from our samples with fragments taken from the ship (deck and hull, respectively green and red) and the net frame (yellow) (Fig. 4). FTIR spectra showed that these paints were indeed derived from the hull, deck and net; red paint fragments from the hull were the most abundant, showed the largest size variation, and the highest amount of macro-sized fragments. Blue, orange, and gray paint fragments were not present on any external structure of our ship, and we therefore infer that the particles with these colors were already at the ocean surface, originating from other continental sources and/or nautical equipment.

### Epiplastic communities

**Figure 5:**
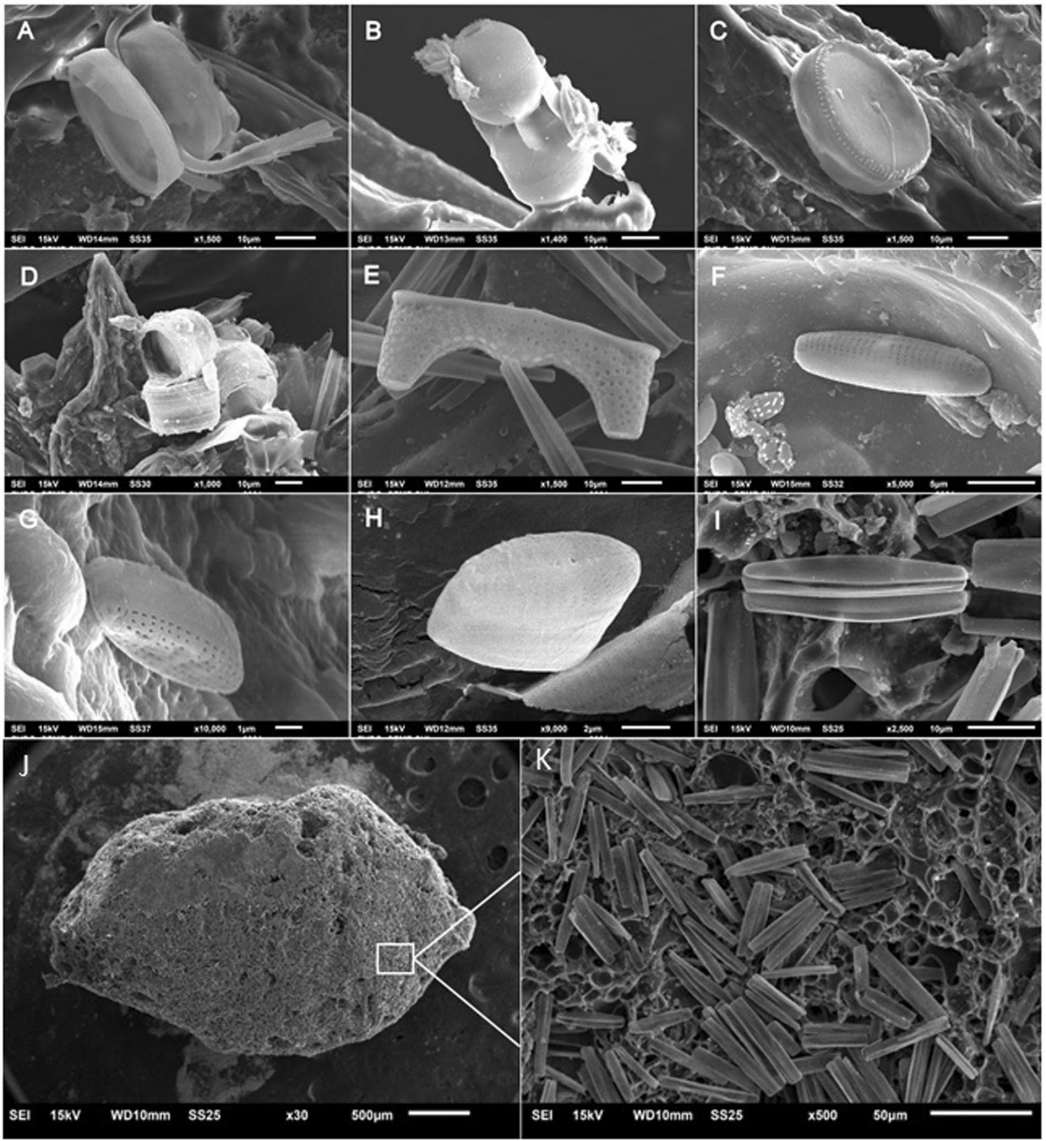
Diatoms found in the Antarctic Plastisphere. (**A**) *Chaetoceros sp*.; (**B**) *Melosira sp*.*;* (**C**) *Thalassiosira* cf. *antarctica* Comber, (**D**) *Thalassiosira sp*.; (**E**) *Eucampia antarctica* (Castracane) Mangin (resting spore valve); (**F**) *Navicula sp*.*;* (**G**) *Navicula* cf. *perminuta* Grunow; (**H**) *Pseudogomphonema* cf. *kamtschaticum* (Grunow) Medlin; (**I**) *Synedropsis sp*.; (**J**) Extruded polystyrene foam piece covered with pennate diatoms of genus *Synedropsis* (overview with 30× magnification); and **K**: 500× magnification of the polystyrene foam for better visualization of microalgae.

**Figure 6:**
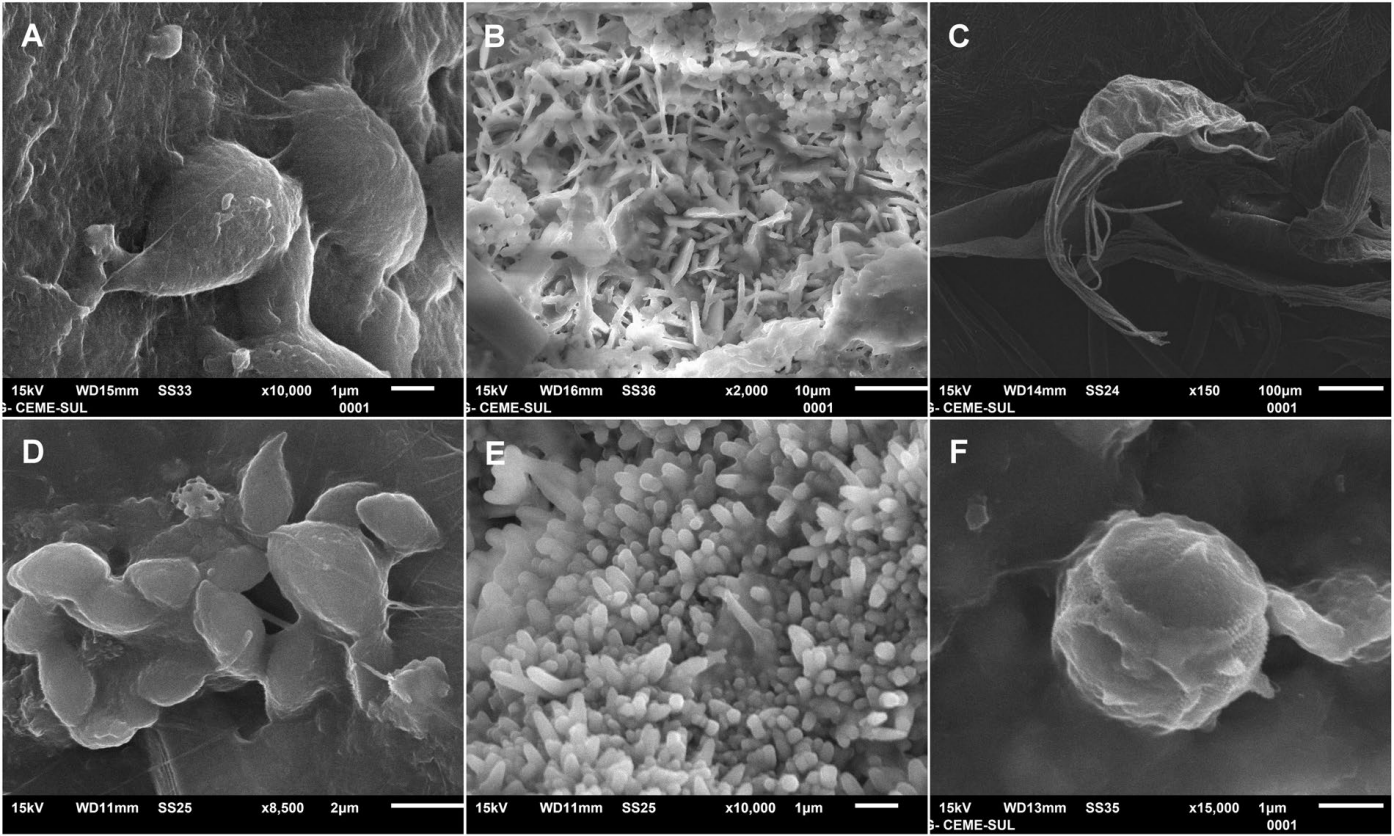
Groups of organisms attached to plastics and paint fragments sampled at surface waters in Antarctica. Coccoid (**A**) and elongated bacterial (**B**) colonies, and marine invertebrate (**C**) adhered to marine plastics; coccoid (**D**) and elongated cells of bacteria (**E**) and *Tetraparma-*like microalgae (Chrysophyceae - Parmales) (**F**) adhered to paint fragments.

Diatoms (centric and pennate) and bacteria were the most abundant groups colonizing plastics and paint chips, but other microalgae and invertebrate groups were also found adhered to the surface of these marine debris. In terms of diatoms, we found species of the genus *Thalassiosira*, *Synedropsis*, *Chaetoceros*, *Navicula*, among others (Fig. 5). We highlight the extruded polystyrene foam fragment completely covered with pennate diatoms of the genus *Synedropsis* (Fig. 5J,K). Coccoid and filamentous bacteria were observed adhered to plastic fragments, lines and spheres (Fig. 6A,B) and in the paint fragments we identified coccoid and elongated cells (Fig. 6D,E), forming large colonies in some cases. We also identified other non-diatom microalgae species, as well as a chrysophyte and invertebrate organisms, in the Antarctic Plastisphere (Fig. 6C,F).

## Discussion

We estimated a mean concentration of 1,794 plastic items.km^−2^ around the Antarctic Peninsula. This concentration lies within the estimated density range of plastic pollution for more than 70% of the world’s oceans: from 1000–100,000 pieces.km^−2^, although closer to the lower end^5^. However, it is a low value when compared to accumulation zones such as the center of the subtropical gyres, e.g. the ‘Great Pacific Garbage Patch’ (with >700,000 pieces.km^−2^)^2,5^ or the Mediterranean (>800,000 pieces.km^−2^)^5^. In any case, considering that Antarctica is an uninhabited area that has only the presence of vessels and research stations, and is an environment with unique biodiversity and ecological relations, this concentration of plastics is alarming and could cause serious environmental damage. The concentration of plastics in surface waters of Antarctica is still poorly known. Barnes & Milner^31^ reported from 0–1 items.km^−2^ of floating marine debris in the surroundings of the Antarctic Peninsula. Microplastics in waters of the Ross Sea have also been described, with similar concentrations (0.0032 to 1.18 particles.m^−3^)^32^ to what was found in the present study, likely due to the proximity between sampling areas. On the other hand, Isobe *et al*.^28^ estimated higher average concentrations for two stations at the eastern portion south of the Polar Front close to the Antarctic continent, with 9.9 × 10^−2^ and 4.6 × 10^−2^ pieces.m^−3^. Additionally, surface waters of the Southern Ocean have been estimated to have from 0.55 to 56.58 g.km^−2^ of plastic fragments^5^. The total average weight of plastics that we found (27.8 g.km^−2^) lies within this estimated weight range of plastics at the surface of the Southern Ocean.

We report a predominance of fragments and lines smaller than 5 mm, showing that the majority of sampled particles are secondary microplastics originating from larger pieces that fragmented due to weathering in the marine environment^22^. In the “sphere” category we found two pellets that could likely have originated from lower latitudes^24^ as there are no local sources of pellets, and according to our model they had probably been in Antarctica for at least seven years if we assume that they had been at the ocean surface for this time. These pellets may also have been retained in ice and/or beaches, being released into the ocean due to melting ice and meteorological events. The large number of nylon line fragments indicates that fishing activities, including IUU (illegal, unreported and unregulated) fishing, occurs around Antarctica and can be a source of plastics for the local environment^24^.

Polyethylene and polypropylene are the two most common plastics found in other parts of the world’s oceans^21,33^, but not around the Antarctic Peninsula. At this region, we found more polyurethane and polyamide, supporting our hypothesis that the main sources of plastics to the Southern Ocean are local, as polyurethane is frequently used in insulation panels, high-resilience foam seating, electrical potting compounds, surface coatings and surface sealants^34^, which are common at research and tourism vessels and research stations. Additionally, polyamide is a characteristic polymer of fishing nets and ropes^23^. The expanded polystyrene pieces found in our samples are also typically used in packaging and fishing gear^35^; fishing-related debris in Antarctic waters have been previously reported by Convey *et al*.^23^ and Ivar do Sul *et al*.^24^. Single-use plastics made of polyethylene and polypropylene were also found in our samples, albeit in smaller numbers, and could represent a problem to the Southern Ocean.

Although the dispersal model showed that the plastics we collected had most likely been south of 58°S for at least seven years or have been released through local sources more recently, it is worth mentioning that they could still have been lost/discarded at lower latitudes and dispersed to and accumulated in Antarctica. Isobe *et al*.^28^ speculated that the absence of relatively “fresh” mesoplastics (from 5 to 20 mm) in their samples could be an indication that the sources of debris are far from the Southern Ocean. However, based on our dispersal model and what has been suggested in other studies^23,24,29,32^, the main sources of plastic at the region, especially around the Antarctic Peninsula, most likely involve local research, tourism and fishing activities. Station 4, which presented sources closer to the continent, is located at a sheltered area close to the peninsula, and could be influenced by coastal currents that retain plastics.

Due to the isolation of the Antarctic Peninsula, and especially the islands around it, marine debris that reaches the coastline can accumulate for many years. These materials can then re-enter the oceans due to wind transport, ice melting, rising/falling sea levels and storms especially smaller particles that are easily carried^23^. Ocean current systems in the western portion of the Antarctic Peninsula, with several convergence zones, can explain the retention of plastics for years within this region, as suggested by Isobe *et al*.^28^ and confirmed by our model. The Antarctic Circumpolar Current, which flows eastwards around the Antarctic continent^36^, may also retain plastic particles within its flow, creating a plastic accumulation zone around the continent that can eventually be dislodged from the system due to events such as storms and vortex formation. At a smaller scale, the Bransfield Current system may form another accumulation zone at the western portion of the Antarctic Peninsula^37^, keeping the plastic particles at the ocean surface of this area for years.

Paint fragments were present at all sampling stations and presented abundance (total n = 2805) of approximately 30 times that of plastics. A similar pattern was observed by Song *et al*.^38^ in surface waters of South Korea’s southern coast, where the authors found around 12 times more paint particles, in different sizes and colors, than plastics. We believe that the paint chips were already at the ocean surface at the time of sampling, since the manta net is lowered using an A-Frame at approximately two meters from the ship and the net mouth does not touch the ship at any time, reducing the chance of contamination. Another indication that the paint fragments were in the ocean is the presence of paint colors that do not belong to our ship (e.g. blue, orange etc.). During sampling, we took care as to avoid the ship’s wake, and considering that we found types of plastics and paint that were not present on our ship, and that biofilm was formed on most fragments, we can infer that most sampled items had already been in the environment for some time. However, it is possible that part of the sampled paint originated from our ship since most were of the colors of the hull (red) and deck (green) (confirmed by FTIR spectra). This could have occurred due to previous shedding, as the ship remains around the Antarctic Peninsula for five to six months every year, or from the net occasionally entering the wake due to wave influence. Although they are denser than seawater, paint particles can float due to water surface water tension^38^. A concerning characteristic of paints used on ships and nautical apparatus is the presence of metals such as Cu, Zn and Pb, and booster biocides used to prevent growth of marine organisms such as sessile invertebrates and algae^39,40^. The impacts of paint fragments in the marine environment may be similar to those of plastics in terms of ingestion and contaminant transfer/biomagnification^41^, as well as attachment and transport of epiplastic organisms (Fig. 6D–F).

Our results showed that plastic and paint fragments in Antarctic waters are a substrate for several species, with the identification of a variety of organisms living on the surface of marine plastics. We found more diatoms when compared to other taxonomic groups, which may be due to the sample preservation method, since freezing and rapid dehydration of the material may have ruptured cells or delicate structures of epiplastic organisms. Epibenthic diatoms (e.g. from genus *Synedropsis*; Fig. 5) were common on floating plastics, which can increase the dispersal rates of these organisms and possibly alter the functioning of the system and compromise ecological relations. Arias-Andres *et al*.^20^ showed that epiplastic organisms influence organic matter cycles in aquatic environments; this could also occur in Antarctica. In addition, although our model showed that it is unlikely that particles arrived from lower latitudes in the last seven years, we cannot discard the risk of bioinvasions resulting from the transport of epiplastic organisms from lower latitudes, or between different Antarctic biogeographic regions. Such risk is concerning for the biodiversity of the Southern Ocean, which is currently suffering from the invasion of species such as the crab *Hyas araneus*^42^ and the mussel *Mytilus gallo-provincialis*^43^. Environmental changes such as ocean acidification and sea surface temperature increase can lead to a greater chance of non-native species reaching and settling in Antarctica via plastics^44,45^. By growing on and interacting with marine plastic, the organisms of the Plastisphere can become contaminated and transfer these contaminants to the organisms that ingest colonized plastics^46^. Epiplastic organisms could also impact the microflora of consumers, since infectious organisms may reach their hosts through plastic ingestion^12,14^. Studies on the microbial communities of marine plastics are still relatively recent, with central issues being focused on the colonization processes, diversity and stability of these communities^47^. Although SEM allows a detailed view of the surface of plastics, it is limited in terms of taxonomic resolution of organims^47^. This reinforces the need for studies using alternative identification tools, such as environmental DNA sequencing (i.e. metagenomic analyses), to characterize the Antarctic epiplastic communities, better revealing their components and ecological impacts.

As previously mentioned, marine plastic pollution can have several effects on the Antarctic ecosystem^23^. The interaction of marine organisms with plastics in Antarctica has already been described in some studies, which show that entanglement and ingestion affect different species of mammals and birds at the region^24^. The shallow waters of the Bransfield and Gerlache straits, where some sampling points were located, are nursery areas for organisms such as krill, a key component of the Antarctic food web^48^. Considering that most of the plastics sampled in this study fell into the ‘microplastics’ category, they could be ingested by and impact krill, as well as other primary consumers, affecting the marine trophic web^49^. Waller *et al*.^29^ highlight that microfibers from synthetic clothes washed at research stations and vessels enter Antarctic waters, especially due to inadequate waste treatment systems and limited on-site inspection. We did not detect any microfibers due to our net’s mesh size, but reaffirm from personal observation that wastewater may be a large source of microplastics at the area. Persistent organic pollutants used in the production of or adsorbed to marine plastics (and paint fragments), which are especially concentrated on microplastics, can have serious consequences such as alteration of growth and reproductive hormones, oxidative stress and reduction of fertility^46,50,51^. The ingestion of plastics by marine organisms has also been demonstrated to reduce energy reserves^9^, potentially leading to their death. Another potential impact of marine plastics occurs during the physical and chemical breakdown of the polymer chain, when carbon is released and transformed into CO_2_^52^, which can contribute to the creation of an anoxic environment and ocean acidification. Finally, plastic degradation can release other greenhouse gases such as methane and ethylene^53^, possibly contributing to climate change.

The results obtained here show that the abundance of plastics in Antarctica is not comparable to high concentration areas such as the center of subtropical gyres or highly urbanized coastlines. However, due to the unique characteristics of this environment, it could be highly sensitive even to low levels of this type of pollution. Plastic pollution in the Southern Ocean has been described since the 1980s, with several studies raising concern regarding this issue^24^, but in accordance with the global scenario, little has been done to effectively reduce the amount of plastics entering the Antarctic environment. If the prevention and mitigation of plastics continues to lag behind its production and inadequate management, we can expect increasing accumulation of plastic waste at the region, since plastic debris from local sources can be retained in Antarctica for long periods due to the relatively closed system created by the Antarctic Circumpolar Current.

The abundance of plastic found at a remote and theoretically pristine region such as the Antarctic Peninsula shows the extent of human influence and the potential irreparability of its impacts on the oceans, reasserting an urgent need for decreasing the production and consumption of plastics, and increasing adequate discard and management practices. The concentration of plastics in Antarctic surface waters reinforces that, despite efforts to limit human use, this region is not exempt from marine plastic pollution. This is possibly due to the lack specific measures and enforcements for solid waste treatment at the area. Antarctica is a world heritage site and one of the most productive oceanic regions on planet, with unique biodiversity, which highlights the importance of elaborating and adopting strategies to conserve this environment. The abundance of paints from nautical vessels/apparatus shows that even the limited scientific and tourist activities are a potential source of pollution in Antarctica. We call for urgent action to avoid plastic and paint fragment inputs to the Southern Ocean, with the implementation of adequate waste management and treatment. We suggest that environmental awareness initiatives with tourists, researchers, ship crews and fishers that use areas around Antarctica be expanded and obligatory. Additionally, further studies should be conducted at the region to increase our understanding of the impacts of plastics to the Antarctic ecosystem, e.g. in terms of entanglement, ingestion and contaminant uptake by marine animals. Finally, a more detailed description of epiplastic communities in the Southern Ocean is fundamental to understand the impact of these organisms on the local and global marine environment.

## Methods

### Sampling

**Figure 7:**
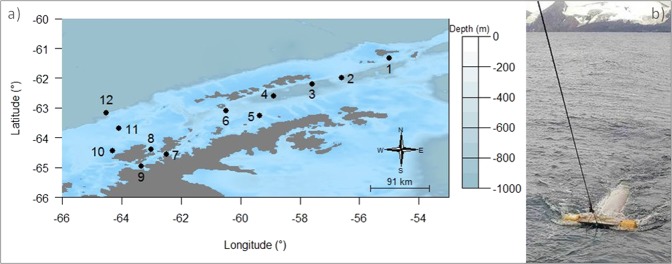
Sampling area of marine plastics in surface waters of twelve points off the Antarctic Peninsula (**a**), using a manta net (**b**). The map was created using the *marmap* package and *getNOAA*.*bathy* function on *R* 3.5.0 (https://www.r-project.org).

In February 2017, during the XXXVI Antarctic Operation and 7^th^ expedition of project “Biological Interactions in Marine Ecosystems off the Antarctic Peninsula Under Different Impacts of Climate Change” (INTERBIOTA), samples were collected in surface waters, at the water-air interface, around the Antarctic Peninsula. These samples were taken at 12 points, between latitudes of 61° and 64°S, using a manta net with a 100 cm × 21 cm mouth and a 330 μm mesh (Fig. 7). At each point, the net was lowered carefully with a large A-frame at approximately 2 m from the windward side of the ship, and was trawled at a speed of 2.5–3.5 knots for between 15–55 minutes. After each trawl, the contents of the collection cup were placed in an aluminum bag and frozen at −40 °C for posterior sorting and analysis. At the start and end of each trawl we noted the geographical coordinates, time, sea state (evaluated by the ship’s officer in charge), local depth and wind speed. The trawled area of each point was calculated based on trawl velocity (considering 1 knots as 0.514 m.s^−1^; *trawl vel*), time (seconds; *t*) and the manta net width (1 meter), and expressed by the equation:

$$Area=trawl\,vel\ast \,t\ast \,{1}$$

The sampled volume was calculated considering the trawled area and full submersion (i.e. 21 cm depth) of the posterior end of the manta net.

### Plastic count and characterization

In the laboratory, we thawed the sampled material of each point separately, and placed it in a sterile container filled with salt water (salinity 35, temperature ~4 °C) for manual separation of floating plastic pieces and biomass^35^. Plastics were identified by naked eye (lower detection limit of approximately 500 microns) by a trained observer (ALdFL) picked up using forceps, and quantified and measured over their largest cross-section (total length) using a digital caliper. In terms of size, plastics were classified as microplastic (<5 mm), mesoplastic (5–200 mm) or macroplastic (>200 mm) (adapted from Eriksen *et al*., 2014)^5^. Each item was weighed with a digital scale (precision of 0.00001 g) and also classified according to format (fragment, line, and sphere), flexibility (rigid, flexible) and color. Polymer composition was determined for 28 plastic pieces selected randomly, through Fourier Transform Infrared Spectroscopy (FTIR) with an equipment SHIMADZU, model Prestige 21, using a diffuse reflectance module, 24 scans and 4 cm^−1^ resolution. To estimate the concentration of plastics at the sea surface, the number and weight of plastics found in the trawled areas was extrapolated to items.km^−2^ and to g.km^−2^ to each sampling point, respectively. The total average concentration and weight of plastics were also calculated. Paint fragments were not included in the concentration analyses and other statistics, being characterized only by color, weight and size classes, as proposed by Song *et al*.^38^ (see section on paint particles in the results). Paint was identified by: (1) visual characteristics of the particle, which were thin, flat and flexible paint chips; and (2) FTIR spectra of 21 samples of paint chips, which indicated their primary polymer as polyurethane, commonly used in paint production.

### Analysis of epiplastic communities

For evaluation of epiplastic communities through Scanning Electron Microscopy (SEM), items from different sampling points were selected from categories ‘fragment’ (n = 8), ‘sphere’ (n = 3) and ‘line’ (n = 3), in a total of 14 plastic pieces. Eight paint fragments were also selected for this analysis, totalizing 22 items. Before SEM, plastic and paint fragments were dehydrated in absolute ethanol (Reagent-grade, MERK). The items were fixed to an aluminum sheet with carbon tape and coated with a 20–30 nm gold layer. The epiplastic organisms were observed using a JEOL microscope (JSM 6610LV, JEOL, Tokyo), operated at 10–20 kV at a working distance of 10–26 mm. For each fragment, the whole item was imaged at a magnification of 25x, followed by imaging at different magnifications (20× to 40,000×) to better record the diversity of organisms. A total of 100 images were evaluated, and the identified individuals were grouped into taxonomic groups (i.e. diatoms, bacteria, etc.) based on morphology, and we attempted to identify the organisms at the lowest possible taxonomic level with the aid of experts of each group.

### Data analysis

To determine if environmental parameters influenced the abundance of plastics at each sampling point, linear regressions were performed between plastic concentration and the parameters local depth, sea state and wind speed, recorded during sampling. To evaluate diversity of plastic colors between the sampling points, we used Shannon’s index, and to check if there was dominance of any colors between the points, we used Simpson’s index. All statistical analyses were done in R (R Development Core Team, version 3.5.0), using the *car* and *vegan* packages.

### Dispersal model

Virtual particles were tracked using the OceanParcels framework^54^ in surface velocity fields from the HYCOM + NCODA Global 1/12° Analysis^55^, on which Stokes drift was added from WaveWatchIII^56^. At each of the 12 locations, 100 virtual particles were released on the day of sampling, and then tracked back in time for seven years, with output stored daily. To simulate subgrid scale motion, a Brownian diffusion of 10 m^2^/s was added. The code for these simulations is available at https://github.com/OceanParcels/AntarcticPeninsulaPlastic.

## Supplementary information

Supplementary Material

## Data Availability

The datasets generated during and/or analyzed during the current study are available in FIGSHARE (10.6084/m9.figshare.7491641) and from the corresponding author upon reasonable request.
