# Supplementary material for: Plastics in sea surface waters around the Antarctic Peninsula
Source: Sci Rep. 2019 Mar 8;9:3977. doi: 10.1038/s41598-019-40311-4 (PMC6408452; doi:10.1038/s41598-019-40311-4)

Plastics in sea surface waters around the Antarctic Peninsula

Ana Luzia de F. Lacerda*, Lucas dos S. Rodrigues, Erik van Sebille, Fábio L. Rodrigues, Lourenço Ribeiro, Eduardo R. Secchi, Felipe Kessler, Maíra C. Proietti

Supplementary Information

Figure S1. Linear regression between the abundance of plastics at each sampling point and sea state (a), wind speed (b) and local depth (c); no statistically significant correlation was observed ($p > 0.05$).

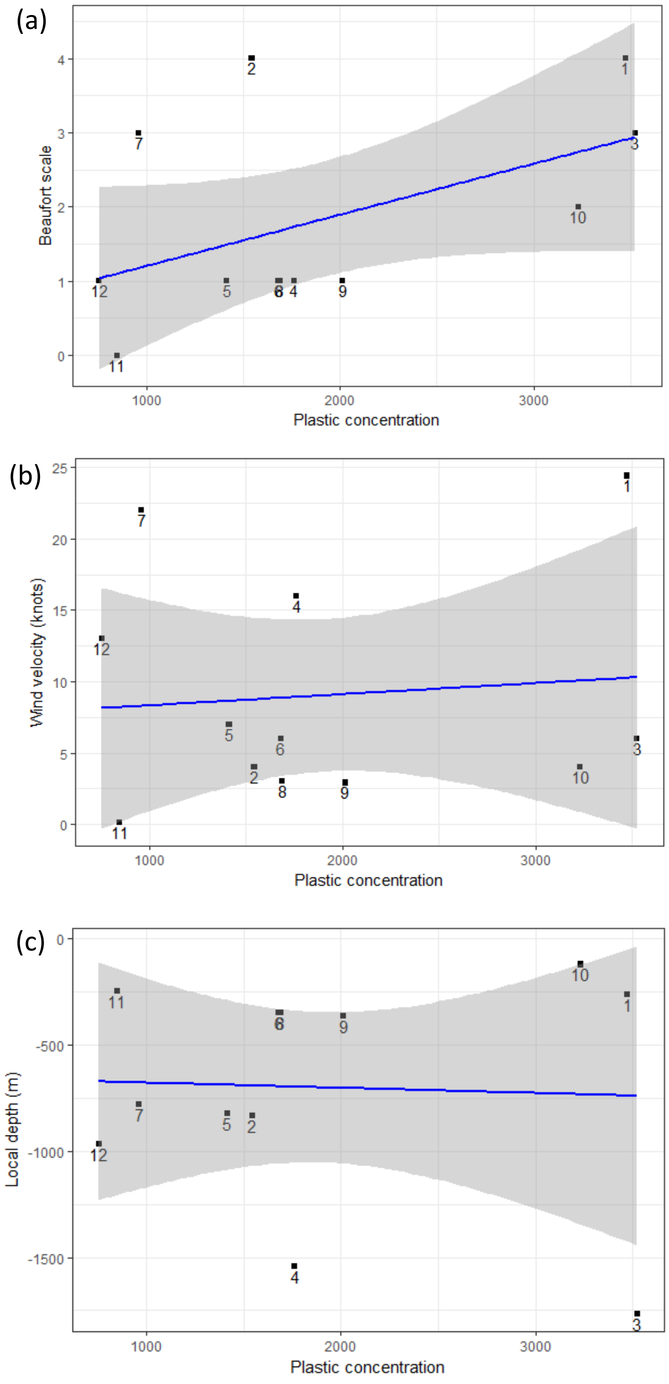

Supplement: Supplementary file 1 — Supplementary Material [file 41598_2019_40311_MOESM1_ESM.pdf]
